# Lymphodepleting chemotherapy practices and effect on safety and efficacy outcomes in patients with solid tumours undergoing T cell receptor-engineered T cell (TCR-T) Therapy: a systematic review and meta-analysis

**DOI:** 10.1007/s00262-022-03287-1

**Published:** 2022-10-31

**Authors:** Kathryn Owen, Ramy Ghaly, Kyrillus S. Shohdy, Fiona Thistlethwaite

**Affiliations:** 1 ATMP Master Programme, The University of Manchester, Manchester, UK; 2 Department of Internal Medicine, University of Missouri-Kansas City, Kansas City, MO USA; 3 Experimental Cancer Medicine Team, The Christie NHS Foundation Trust, Wilmslow Road, Manchester, M20 4BX UK; 4 Division of Cancer Sciences, The University of Manchester, Manchester, UK

**Keywords:** TCR-T cell, Solid tumours, Lymphodepletion, Conditioning chemotherapy, Safety, Efficacy

## Abstract

**Background:**

T cell receptor-engineered T cell (TCR-T) therapy has shown promising efficacy in advanced solid tumours. Lymphodepleting (LD) chemotherapy improves TCR-T cell therapy efficacy but is associated with significant toxicities. Evidence is sparse regarding the optimum LD regimen for TCR-T cell therapy in solid tumours.

**Methods:**

A systematic review was conducted of interventional, prospective clinical trials describing LD practices prior to TCR-T cell therapy in patients with advanced solid tumours. The objective was to define LD regimens administered prior to TCR-T cell therapy and their effects on specific safety and efficacy outcomes in this patient population.

**Results:**

Searches returned 484 studies, 19 (231 patients) met the eligibility criteria. Cyclophosphamide (cyclo) 60 mg/kg daily (2 days), plus fludarabine (fludara) 25 mg/m^2^ daily (5 days) was the most common LD regimen (38% of studies). Higher dose LD regimens were associated with increased pooled incidence rates of febrile neutropaenia compared to low dose (0.64, [95% Confidence interval (CI): 0.50–0.78], vs. 0.39 [95% CI: 0.25–0.53], respectively) but were not significantly associated with higher objective responses (odds ratio: 1.05, 95%CI: 0.60–1.82, *p* = 0.86). A major shortfall in safety data reporting was identified; determination of LD regimen effects on many safety outcomes was not possible.

**Conclusion:**

Standard consensus guidelines for the design and reporting of adoptive cell therapy (ACT) studies would facilitate accurate risk–benefit analysis for optimising LD regimens in patients with advanced solid tumours.

**Supplementary Information:**

The online version contains supplementary material available at 10.1007/s00262-022-03287-1.

## Introduction

Patients with advanced solid tumours relapsed or refractory to standard of care therapies have a poor prognosis, with few further treatment options. Adoptive T cell therapy (ACT) has shown promising efficacy in various solid tumour types [1, 2]. Since early trials of tumour infiltrating lymphocytes (TILs), T cell therapies have evolved and include chimeric antigen receptor-T (CAR-T) cell and T cell receptor-engineered T (TCR-T) cell therapies [3]. TCR-T cells target a greater variety of antigens than CAR-T cells, which recognise only cell surface antigens, so may have greater efficacy in solid tumours, supported by response rates from recent clinical studies [4–6]. To date, no TCR-T cell therapies are licensed, albeit a positive risk benefit balance has been demonstrated in melanoma and other solid malignancies [6–9].

An essential requirement for ACT efficacy is the in vivo expansion and persistence of T cells once reinfused [1, 10, 11]. Clinical trial data show that lymphodepleting conditioning (LD) regimens consisting of cyclophosphamide (cyclo) and fludarabine (fludara) prior to cell reinfusion improves T cell in vivo expansion and persistence [12, 13].

ACT is associated with specific toxicities, including cytokine release syndrome (CRS) and immune effector cell-associated neurotoxicity syndrome (ICANS) [14]. Neurological adverse reactions are common to CAR-T cell therapy and cyclo/fludara dosing, so when given together, the lack of randomised controlled studies render causality assignment difficult for neurological toxicities [15].

Most safety and efficacy outcome data for LD regimens derive from CD19 CAR-T cell studies in haematological malignancies, which may not apply to TCR-T cell therapy in solid tumours. Differences include the direct access of CAR-T cells to tumour, high local concentrations of immune effector cells implicated in CRS, and neurological toxicity due to ‘on target, off tumour’ toxicity in haematological malignancies [16]. LD in haematological malignancies results in direct reduction of the malignant cell population. Bone marrow reserve is often lower than for patients with solid tumours, so rates of cytopaenias and bone marrow aplasia may differ for a given LD regimen [12]. Solid tumours inhabit an immunosuppressive microenvironment, the aim of LD being to reduce immunosuppressive cells.

Given the pivotal importance of LD in ACT, lack of randomised controlled clinical trials comparing LD regimens, and scarcity of TCR-T cell trials in solid tumours, leading to a lack of consensus regarding optimal regimens, a systematic review may help fill this knowledge gap. This review will summarise the lymphodepleting practices employed prior to TCR-T cell therapies and effects on safety and efficacy outcomes in patients with relapsed, refractory solid tumours, participating in interventional, prospective clinical studies.

## Methods

### Literature search and eligibility criteria

This review was written as part of a Master of Science (M.Sc.) thesis, planned prospectively with the supervisory committee and registered with the University of Manchester. It is reported per the Preferred Reporting Items for Systematic Reviews and Meta-analyses, ‘PRISMA-P’ guidelines [17, 18]. MEDLINE, Embase, ACP Journal Club, Cochrane Database of Systematic Reviews, Database of Abstracts of Reviews of Effects, Cochrane Central Register of Controlled Trials and Web of Science databases were searched, and literature retrieved, from 28 June to 4 August 2021. The search covered from the date of literature inception to 28 June through 4 August 2021. The search strategy used keywords (e.g. TCR, TCR-engineered T cells) and controlled vocabulary (e.g. receptors, antigen, T cell) related to study objectives, with no restrictions on the date of publication (full search strategy examples, Supplementary Tables 1a and 1b).

Studies were screened by a single reviewer, KO, using prespecified eligibility criteria, following the PICOTS (Population, Intervention, Comparison, Outcome, Timing, Study design) framework (Supplementary Table 2). References of related and systematic reviews were cross-referenced with the search strategy to include additional relevant studies. ClinicalTrials.gov was searched for updated results or missing methodological details not included in the original articles/abstracts.

### Objectives

These were to define LD regimes administered prior to TCR-T cell infusion and effects on safety and efficacy outcomes in patients with solid tumours. LD was defined as a treatment to reduce the population of circulating lymphocytes, prior to infusion of TCR-T cells, including chemotherapy, radiotherapy and/or any other method specified. High-dose LD regimens included total doses of cyclo ≥ 120 mg/kg and fludara ≥ 100 mg/kg^2^. Specific safety outcomes included adverse events (AEs) of cytopaenias, bone marrow aplasia, infections, CRS, neurotoxicity, and Graft-versus-host disease (GvHD). Specific efficacy outcomes included objective response rate (ORR), defined by the Response Evaluation Criteria in Solid Tumours (RECIST) Guideline, v1.1.[19].

### Risk of bias

No specific tool exists for risk of bias assessment of single-arm interventional studies; therefore, Grigor and colleagues’ risk of bias tool for interventional study designs was used to assess the quality of included studies [4, 20] (Supplementary Table 3). Studies that met all eligibility criteria but had a high risk of bias were included in the review but excluded from data analyses per the Cochrane Handbook recommended the best practice to reduce the risk of reporting bias [21].

### Data synthesis and analysis

All analyses were conducted on an intention-to-treat population. Heterogeneity across subgroup analyses was tested by I-square (I^2^) and Chi-square tests. The Chi-square test measures the existence of a significant heterogeneity, whilst I^2^ quantifies the magnitude of heterogeneity in the effect size. If both tests showed low heterogeneity, the fixed effect model (Mantel–Haenszel method) was applied. If a high degree of heterogeneity was observed, a random-effect model was applied. A sensitivity analysis was conducted to identify sources of heterogeneity using “one-out” approach [22]. Single studies that lead to a decrease of I^2^ < 25% were reported if identified.

The analysis was conducted in STATA 16.0 [48]. The *metaprop* command was applied to calculate pooled incidence rates of AEs and ORR. To test the impact of the ordered values of the dose level categories of cyclo or fludara on the objective response status, *nptrend* command was used in STATA to run the Cuzick test.

## Results

### Characteristics of the included studies and participants

Systematic searches retrieved 484 potentially relevant citations; 433 were excluded based on abstract/title. 51 studies remaining were read in full, and 12 articles (185 patients) and 7 abstracts (46 patients) met the eligibility criteria (Supplementary Fig. 1). Two articles [23, 24] reported all outcome data for cohort 1, and efficacy data only for all 4 cohorts, respectively, for the same study. The four cohorts received different LD regimens. Data for all 4 cohorts were updated in a recent abstract [25]. Hence, both articles and abstract were included. Two abstracts [26, 27] reported data for two patient cohorts respectively, separately, receiving different LD regimens, within the same study; hence, both are included. All studies administered autologous T cells. No paediatric patients were included (Table 1).

**Table 1:** Characteristics of the included studies and participants

Articles First Author, Year	Trial phase	No. of patients dosed, No. of prior therapies (median, range)	Age, years (median, range)	Cancer type, target	Follow-up post-TCR-T cell infusion
D’Angelo 2021**[27]	I/II	10, NR	NR	MRCLS NY-ESO-1	Safety, NR Efficacy, ORR 12 wks
Nagarsheth 2021[7]	I/II	12, 4 (3–7)	47 (31–65)	Vulv, cerv, anal, SCCHN HPV-16 E7	Safety, day 40 Efficacy, 8 months
Hong D. 2020 [28]	I	5, NR	NR	Ov, SCCHN, MRCLS, oes MAGE-A4 + CD8α co-receptor	NR
D’Angelo 2020*[25]	I/II	45, NR	NR	SS NY-ESO-1	Efficacy, cohorts 1–4, median 480/278/605/643 days resp.
Nowicki 2019 [29]	I	10, 3 (2–7)	42 (24–66)	SS, osteo, mel, lip, NSNY-ESO-1	Efficacy, (ORR) day 90
Doran 2019 [8]	I/II	12, 2 (1–6)	50 (32–70)	Cerv, anal, vag, SCCHN HPV-16 E6	Safety, NR Efficacy, 12 months
Ramachandran 2019* [24]	I/II	45, NR	NR	SS NY-ESO-1	Safety, NR Efficacy, cohorts 1–4, median 480/278/605/643 days resp.
Hattori 2019 [30]	I	9, NR	NR	SS NY-ESO-1	NR
Butler 2019 [31]	Ib	9, NR	NR	Endom, ov, SS, mel NY-ESO-1	NR
D’Angelo 2018*[23]	I/II	12, 2 (1–4)	29 (18–51)	SS NY-ESO-1	Efficacy, median day 480
Moore 2018 [32]	I	3, 4 (1–4)	56 (41–66)	Mel Tyrosinase	Safety, day 10 Efficacy, median 255 (range 42–523) days
Stadanlick 2018**[26]	I/II	10, 4 (NR)	48 (NR)	MRCLS NY-ESO-1	Safety, NR Efficacy, 4–20 wks
Hong D. 2018 [33]	I	3, NR	NR	MAGE-A4 positive (any)	NR
Lu 2017 [9]	I/II	17, 4 (2–12)	52 (25–66)	Mel, SS, osteo, BC, cerv, anal, uro, oes MAGE-A3	NR
Kageyama 2015 [34]	I	10, 2 (1–3)	61 (43–73)	Oes MAGE-A4	Safety, day 35 Efficacy, day 63? Unclear
Robbins 2015 [6]	II	45, SS 3.5 (2–8), Mel 1 (1–3)	SS 39 (19–65) Mel 51 (30–65)	SS, Mel NY-ESO-1	Efficacy, 3–5 years
Chodon 2013 [35]	II	13, 1 (0–7)	50 (40–61)	Mel MART-1	Efficacy, day 90
Morgan 2013 [36]	I/II	9, 5 (3–8)	56 (21–71)	Mel MAGE-A3	NR
Hong J. 2010 [37]	I	9, NR	40 (25–56)	Mel + brain metastases Gp100/MART-1	Unclear

*HPV* Human papilloma virus, *SS* Synovial sarcoma, *mel* Melanoma, *BC* Breast cancer, *cerv* Cervical, *NS* Nerve sheath tumour, *oes* Oesophageal, *ov* Ovarian, *lip* Liposarcoma, *SCCHN* Squamous cell carcinoma of head and neck, *uro* Urothelial, *vag* Vaginal, *vulv* Vulval, *MRCLS* Myxoid/round cell liposarcoma, *endom* Endometrial, *NR* Not reported

^*^D’Angelo 2020 abstract contains updated dosing, safety and efficacy data for D’Angelo 2018 and Ramachandran 2019 articles (all report the same study)

^**^D’Angelo 2021 abstract reports data for cohort 2, Stadanlick 2018 abstract for cohort 1 of the same study

### Risk of bias and quality assessment of included studies

Risk of bias is summarised across all studies (Supplementary Fig. 2), with each risk of bias item assessment for individual studies presented (Supplementary Fig. 3). All studies were single-arm, non-randomised, and non-controlled, and therefore had a high risk of interpretation bias. Methods for measurement of safety and efficacy outcomes were not clearly stated in 40% of studies. Every study lacked an independent, blinded assessor for outcomes and showed a high risk of bias regarding patient recruitment. 40% of studies clearly reported AEs, with 95% of studies reporting ORR, demonstrating a strong reporting bias for favourable versus unfavourable outcomes. The follow-up period for data cut-off, reported in 53% of studies, was highly variable, potentially affecting data reporting (AEs resolution, delayed/chronic AEs, delayed responses, response duration, overall survival).

### Lymphodepleting regimens prior to TCR-T cell therapy

Ten different dose levels were administered as LD chemotherapy across the 21 studies/cohorts. Cyclo 120 mg/kg (60 mg/kg daily, 2 days), plus fludara 125 mg/m^2^ (25 mg/m^2^ daily, 5 days), given in 75% of cases on days − 7 to − 6 (cyclo) and − 5 to − 1 (fludara) was the most commonly used in 8/21 (38%) studies/cohorts. Cyclo monotherapy without fludara was used in 14%. One study (5%) used no lymphodepletion [34].

TCR-T cell therapy targeted 7 different tumour antigens, with NY-ESO-1 the most frequent, doses ranging from 1 × 10^7^ to 13 × 10^10^ cells. Four studies included separate cohorts receiving escalating doses of TCR-T cells. Interleukin-2 (IL-2) was used in 9/19 studies. Other adjunct therapies included colony stimulating factors, ipilimumab, and peptide antigen/primed dendritic cell vaccination (Supplementary Table 4).

### The pooled incidence of adverse effects

**Fig. 1:**
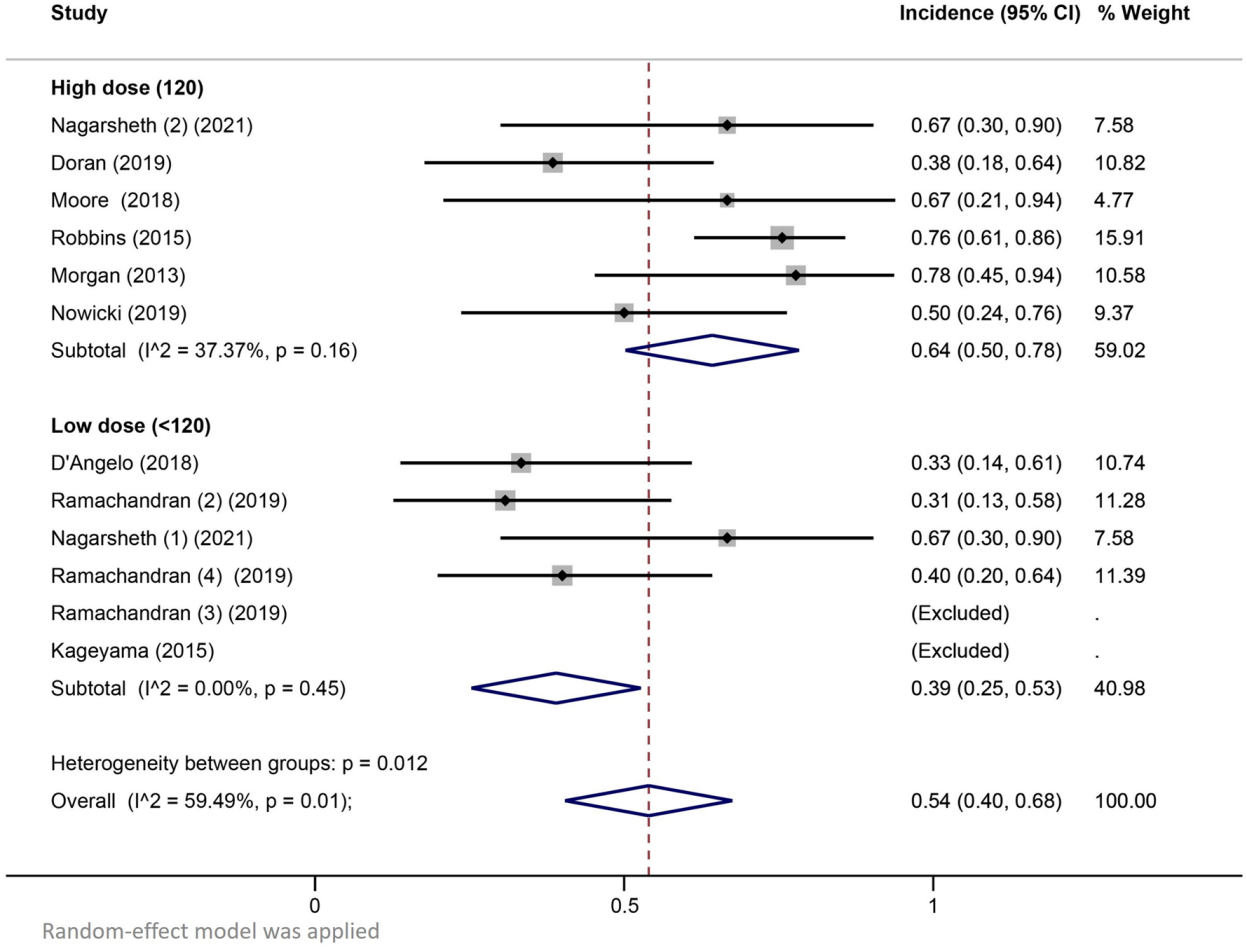
Pooled incidence rates of febrile neutropaenia according to lymphodepleting chemotherapy dose

Nine studies reported detailed adverse effects (AEs) data. Pooled incidence rates showed a trend for febrile neutropaenia and Grade ≥ 3 anaemia occurring more frequently among patients receiving high-dose LD regimens (Table 2). Pooled incidence rates of febrile neutropaenia among patients receiving high-dose LD regimens and low-dose LD regimens were 0.64 (95% CI 0.50–0.78) and 0.39 (95% CI 0.25–0.53), respectively (Fig. 1). Significant heterogeneity was observed in the high-dose subgroup and overall analyses of febrile neutropenia (I^2^ 37.37% and 59.49%, respectively).

**Table 2:** Pooled incidence rates of grade ≥ 3 cytopaenias

Grade ≥ 3 cytopaenia	LD regimen	Rate	95% CI
Anaemia	High dose	0.81	(0.71–0.90)
	Low dose	0.58	(0.28–0.87)
	Overall	0.65	(0.48–0.83)
Febrile neutropaenia	High dose	0.64	(0.50–0.78)
	Low dose	0.39	(0.25–0.53)
	Overall	0.54	(0.40–0.68)
Neutropaenia	High dose	0.88	(0.76–1.0)
	Low dose	0.78	(0.67–0.89)
	Overall	0.82	(0.74–0.90)
Thrombocytopaenia	High dose	0.66	(0.35–0.97)
	Low dose	0.64	(0.48–0.81)
	Overall	0.64	(0.49–0.79)
Lymphopaenia	High dose	1.00	NR
	Low dose	0.57	(0.42–0.73)
	Overall	0.6	(0.47–0.73)

Based on the lack of reported safety data, it was not possible to determine the effect of LD regimen on AEs of CRS, neurotoxicity, infection, or GvHD. (Supplementary Tables 5, 6).

### Objective response rate (ORR)

**Fig. 2:**
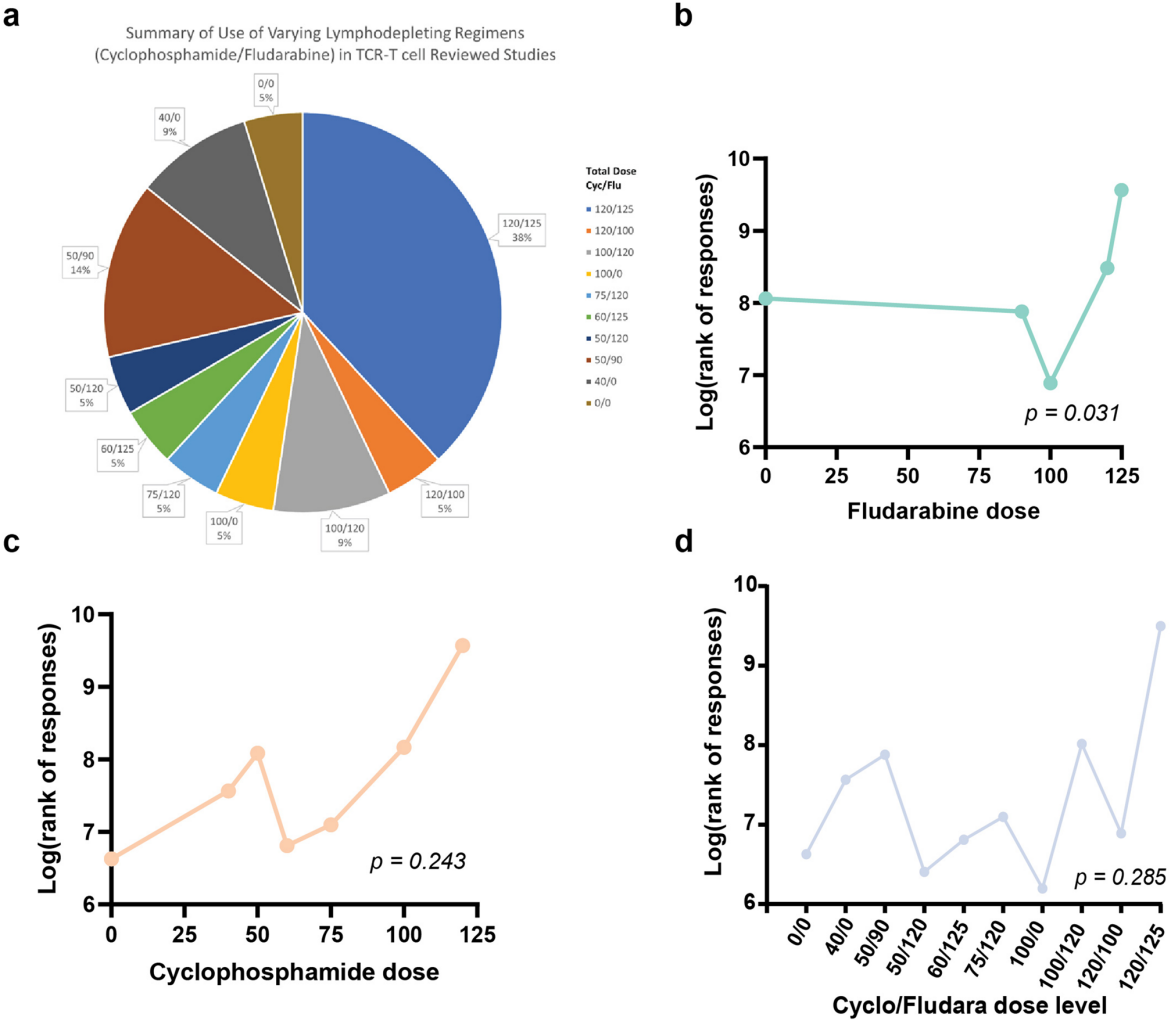
Impact of ordered dose levels of fludara and cyclo either alone or combined on the objective response rate. **a** Ten dose levels of cyclophosphamide and fludarabine were used across the included studies with seven different dose levels of cyclophosphamide and five for fludarabine. **b** Higher dose levels of fludara were associated significantly with a higher probability of objective responses, represented on the y-axis as the natural log of the sum of ranks of responses computed by Cuzick’s test. **c** Dose levels of cyclophosphamide were not significantly associated with a higher probability of objective responses. **d** Dose levels of cyclophosphamide and fludarabine were not significantly associated with a higher probability of objective responses

The ORR ranged from 0 to 67% (Supplementary Table 7). Using the reported aggregate objective response rates, the pooled ORR for low-LD-dose and high-LD-dose cohorts were 0.31 (95% CI 0.15–0.46) and 0.36 (95% CI: 0.26–0.45), respectively (Supplementary Fig. 4). We were able to extract the individual cyclo and fludara doses with the corresponding response status for 231 patients. High LD dose was not associated with a higher likelihood of objective response (odds ratio: 1.05, 95%CI: 0.60–1.82, *p* = 0.86). We tested the impact of ordered dose levels of fludara and cyclo either alone or combined on the objective response (Fig. 2a). Only higher dose levels of fludara were associated with a significantly higher probability of objective responses (*p* = 0.03) (Fig. 2b). This was not significant with cyclo (*p* = 0.24) nor combined cyclo/fludara dose levels (*p* = 0.28) (Fig. 2c,d).

## Discussion

This systematic review identified a major shortfall in reporting of data from TCR-T cell clinical trials in patients with solid tumours. Firstly, LD regimens were not sufficiently detailed in 9 of the potentially suitable 51 studies, increasing the potential for bias (missing data). Secondly, reporting of key, life-threatening AEs of CRS, neurological toxicity, infection, and GvHD was frequently inconsistent or missing.

For studies split into cohorts receiving different LD regimens, data were reported for the entire study population, not per cohort. Where reported, criteria used for CRS definition and severity were rarely cited. Some authors reported the three components of CRS (fever, hypotension, and hypoxia) as separate AEs [7]. Taken together, the lack of AE data reporting makes the correlation of LD regimens with safety outcome data, including the true rate of CRS for a given LD regimen, difficult. However, a trend for an association between high-dose LD regimens and the incidence of febrile neutropaenia and grade ≥ 3 anaemia was observed, albeit heterogeneity between high- and low-dose LD groups was significant. Dudley and colleagues first demonstrated that a LD regimen containing total doses of cyclo 120 mg/kg and fludara 100 mg/m^2^ significantly increased the efficacy of TIL therapy in metastatic melanoma, with manageable toxicity [13]. Adoption of this LD regimen in CAR-T cell therapy demonstrated that higher dose cyclo/fludara use was also associated with improved efficacy [38–40] but an increased toxicity burden in both haematological and solid tumours [12]. Consequently, a range of lower cyclo/fludara doses have been studied as LD in ACT therapy in solid tumours [12, 49–51]. For this review, we considered the original standard LD regimens containing total doses of cyclo ≥ 120 mg/kg and fludara ≥ 100 mg/m^2^ as ‘’high dose’’. Cyclophosphamide monotherapy used in 2 of the studies was considered a “low-dose” LD regimen. Higher dose fludara, but not higher dose cyclo nor cyclo/fludara, was associated with a significantly higher probability of objective responses (*p* = 0.03, *p* = 0.24, and *p* = 0.285, respectively) in this review, supported by data from two non-Hodgkin’s Lymphoma studies, where LD regimes containing fludarabine were associated with improved CAR-T cell expansion, persistence and efficacy, compared with non-fludarabine containing regimes [38, 39]. A multivariate analysis of factors affecting progression-free survival (PFS) in a study of CAR-T cells in B cell lymphoma, using low- or high-intensity LD, found that specific cytokine concentrations above the median were associated with improved PFS [40]. PFS was improved in patients receiving high-intensity LD and achieving a favourable cytokine profile, compared with those receiving the same high-intensity LD without achieving a favourable cytokine profile [40], suggesting that the effects of LD regimen on specific cytokine profiles are more important in terms of CAR-T cell efficacy than the intensity of LD. It remains to be seen whether this applies to TCR-T cell therapy in solid tumours, as data for cytokine profiles were not collected in this review.

Included studies employed a range of adjunct therapies. The rates of febrile neutropaenia were slightly lower (range 0–0.4) in the 2 studies reporting G-CSF use [23, 24] than for studies not reporting G-CSF use, but 2 of the cohorts used lower doses of cyclo with no fludara (100/0 and 50/0, [23]). As a potential routine component of TCR-T cell therapy, reporting of G-CSF use may have been omitted, making it difficult to determine its effect on AE incidence. The addition of IL-2 (9/19 studies) does not seem to have significantly influenced efficacy outcomes in this review, but data are confounded by variations in doses of IL-2 (72,000, 500,000, 720,000 units tds), doses of TCR-T cells and LD therapy, and use of other adjunct therapies (DC and peptide vaccinations). For these reasons, and due to inconsistent AE reporting (CRS, neurotoxicity, GvHD), the effect of adjunct therapies on safety and efficacy outcomes is therefore confounded and difficult to interpret.

## Limitations of this review

Peer review of the search strategy may have reduced potential biases. The process of article and abstract selection from systematic searches for inclusion in the review, and quality assessments of the risk of bias for individual studies, were both intrinsically biased, relying on the interpretation and judgement of a single reviewer (KO), with no second reviewer or opportunity for discussion with peers (selection and reporter bias).

This review demonstrated a high level of clinical and methodological heterogeneity between and within studies of TCR-T cells in patients with solid tumours, in keeping with findings from similar systematic reviews/metanalyses of studies in CAR-T cells [4, 41]. Notable was the number of variables changing concurrently within a study and within small patient cohorts. Patients often had different tumour types, LD regimens, TCR-T cell doses or adjunct therapies, so it was impossible to decipher the effect of changing a single variable on an outcome. Therefore, the interpretation of the effect of LD regimen on safety and efficacy outcomes is heavily confounded by multiple factors, some largely outside study control, including patient characteristics (age, performance status, tumour burden at baseline, tumour and off-target expression of target antigen, number/nature of prior therapies, comorbidities, concomitant medications, cyclo/fludara PK), but also TCR-T cell variables (target antigen, cell dose, ‘fitness’[phenotype], persistence and expansion of cells, number of infusions of cells), adjunct therapies (IL-2, dendritic cell or peptide vaccination, immune checkpoint inhibitors) and follow-up periods.

Due to the rarity of TCR-T cell therapy, clinical trial designs should focus on key objectives and outcomes, fixing other variables so that confounding is minimised and the interpretation of an effect on an outcome is as robust as possible. Standardisation of clinical trial designs in TCR-T cell therapy and ACT in general, with a consensus for reporting LD regimen, TCR-T cell dose, adjunct therapies, defined safety/efficacy outcomes, and follow-up times, per the master protocols from the Pan American/World Health Organisations’ Master Protocol for clinical trials in COVID-19, may allow more valid data pooling and maximise available evidence [42]. Consensus should be reached on CRS definition and grading, neurological toxicities, and GvHD and adopted globally.

The risk of bias in the results was very high, i.e. the risk of over- or under-estimation of the true intervention effect (LD regimen) on safety and efficacy outcomes post TCR-T cell therapy. There are specific challenges involved in the production of robust, quality data for risk–benefit analyses of ACT using randomised controlled clinical trials [43]. TCR-T cell therapy is a bespoke product for patients with relapsed/refractory life-threatening disease, with no other suitable treatment options. Few patients are suitable for TCR-T cell therapy; their acquisition, manufacture, storage, shipping, clinical delivery, and patient care are highly complex, with a substantial production failure rate [1]. Together with high costs, these factors preclude larger clinical trials, and the lack of suitable alternative therapies prevents the ethical use of comparator arms.

The primary outcome for this review was to summarise the LD *dosing* regimens prior to TCR-T cell therapy in patients with solid tumours. A more accurate primary outcome would have been a summary of *exposures* of the chemotherapies used and safety and efficacy outcomes. Hepatic and renal function affects cyclo and fludara pharmacokinetics (PK), so for a given dose, there is high inter-patient variability in systemic exposure, despite adjustments for body weight or body surface area (BSA), potentially influencing outcomes [44–47]. Sparse PK sampling during LD may allow a more accurate correlation of cyclo/fludara *exposure* with safety and efficacy outcomes, with optimisation of future LD dosing, and improvement in LD-associated risks and benefits.

## Conclusion

To our knowledge, this is the first systematic review of lymphodepleting practices employed prior to TCR-T cell therapy in patients with relapsed, refractory solid tumours, participating in interventional, prospective clinical studies. Such studies are infrequent, extremely heterogeneous, and the risk of bias for outcomes due to publication, methodological, reporting, and interpretation biases in this review is very high. The most commonly used LD regimen (38% of studies/cohorts) prior to TCR-T cell therapy was 60 mg/kg cyclo and 25 mg/m^2^ fludara, given daily for 2 and 5 days, (total doses 120 mg/kg, 125 mg/m^2^), respectively. Febrile neutropaenia and grade ≥ 3 anaemia tended to occur more frequently among patients receiving high-dose LD regimens. Higher-dose fludara (≥ 100 mg/m^2^) was associated with a significantly higher probability of objective responses, but this did not apply to cyclo nor combined cyclo/fludara at higher doses. Taken together, these data imply that lower doses of cyclophosphamide may be adopted without compromising TCR-T cell therapy efficacy outcomes.

Safety outcome reporting was inconsistent. Standardised TCR-T cell therapy clinical trial designs and data reporting may allow appropriate pooling of data and facilitate the development of robust evidence bases for future optimisation of LD regimens which may reduce morbidity mortality, shorten hospital stay, and reduce intensive care unit admissions, resulting in a longer and better quality of life for patients with advanced solid tumours with no other treatment options.

### Supplementary Information

Below is the link to the electronic supplementary material.

Supplementary file1 (DOCX 2943 kb)
