# Supplementary material for: Lymphodepleting chemotherapy practices and effect on safety and efficacy outcomes in patients with solid tumours undergoing T cell receptor-engineered T cell (TCR-T) Therapy: a systematic review and meta-analysis
Source: Cancer Immunol Immunother. 2022 Oct 31;72(4):805–14. doi: 10.1007/s00262-022-03287-1 (PMC9628360; doi:10.1007/s00262-022-03287-1)

**Supplementary Information file**

**Lymphodepleting chemotherapy practices and effect on safety and efficacy outcomes in patients with solid tumours undergoing T cell receptor-engineered T cell (TCR-T) Therapy; A systematic review and meta-analysis**

**Owen *et al*.**

# **Supplementary Table 1a**

# **Final EMBASE Cochrane search results**

**Database:**
EBM Reviews - Cochrane Database of Systematic Reviews <2005 to July 28, 2021>
EBM Reviews - ACP Journal Club <1991 to July 2021>
EBM Reviews - Cochrane Clinical Answers <July 2021>
EBM Reviews - Database of Abstracts of Reviews of Effects <1st Quarter 2016>
Embase <1974 to 2021 August 03>

| **#** | **Query** |  |
| --- | --- | --- |
| 1 | TCR.mp. [mp=ti, ab, tx, kw, ct, hw, tn, ot, dm, mf, dv, fx, dq] | 44,466 |
| 2 | TCR-engineered T cells.mp. [mp=ti, ab, tx, kw, ct, hw, tn, ot, dm, mf, dv, fx, dq] | 200 |
| 3 | clinical trial*.mp. [mp=ti, ab, tx, kw, ct, hw, tn, ot, dm, mf, dv, fx, dq] | 1,832,333 |
| 4 | clinical study.mp. [mp=ti, ab, tx, kw, ct, hw, tn, ot, dm, mf, dv, fx, dq] | 4,341,519 |
| 5 | 3 or 4 | 5,683,929 |
| 6 | NY-ESO-1.mp. [mp=ti, ab, tx, kw, ct, hw, tn, ot, dm, mf, dv, fx, dq] | 2,127 |
| 7 | MART.mp. [mp=ti, ab, tx, kw, ct, hw, tn, ot, dm, mf, dv, fx, dq] | 4,638 |
| 8 | MAGE.mp. [mp=ti, ab, tx, kw, ct, hw, tn, ot, dm, mf, dv, fx, dq] | 9,730 |
| 9 | (E7 or E6).mp. [mp=ti, ab, tx, kw, ct, hw, tn, ot, dm, mf, dv, fx, dq] | 17,743 |
| 10 | 6 or 7 or 8 or 9 | 33,490 |
| 11 | 1 or 2 | 44,466 |
| 12 | 5 and 10 | 8,312 |
| 13 | 11 and 12 | 226 |
| 14 | limit 13 to english language [Limit not valid in CDSR,ACP Journal Club,CCA,DARE; records were retained] | 226 |
| 15 | limit 14 to human [Limit not valid in CDSR,ACP Journal Club,CCA,DARE; records were retained] | 202 |
| 16 | from 15 keep 3,8,10,12-14,27-30,32,40,44-48,50-52,62-63,65-69,77-78,80,82,86-87,91-92,95,99-100,102-104,112,118,121-122,138,155,160,163,187,198 | 51 |
| 17 | from 16 keep 2-3,5,12,16-17,26,30-31,34-35,44-47,50-51 | 17 |
| 18 | from 17 keep 1-8,10-17 | 16 |

# **Supplementary Table 1b**

# **Final MEDLINE, E-PUB search results**

**Database:**
Ovid MEDLINE(R) and Epub Ahead of Print, In-Process, In-Data-Review & Other Non-Indexed Citations and Daily <1946 to June 28, 2022>

| **#** | **Query** |  |
| --- | --- | --- |
| 1 | adoptive cell therapy.mp. | 1,340 |
| 2 | gene therapy.mp. or Genetic Therapy/ | 76,214 |
| 3 | *T-Lymphocytes/ or exp Receptors, Antigen, T-Cell/ or TCR.mp. | 136,472 |
| 4 | TCR-engineered T cell*.mp. | 122 |
| 5 | 3 or 4 | 136,472 |
| 6 | 1 or 2 | 77,492 |
| 7 | 5 and 6 | 1,707 |
| 8 | lymphodepletion.mp. | 358 |
| 9 | lymphodepleting.mp. | 199 |
| 10 | 8 or 9 | 509 |
| 11 | 7 and 10 | 23 |
| 12 | 5 and 10 | 205 |
| 13 | 12 | 205 |
| 14 | limit 12 to (english language and humans) | 173 |
| 15 | from 12 keep 34,76,101-102,129 | 5 |
| 16 | from 14 keep 28,64,71,88-89,108 | 6 |

| Supplementary Table 2.  Population, intervention, comparison, outcome, timing and study design (PICOTS) breakdown of eligibility criteria | | |
| --- | --- | --- |
| Category | **Inclusion Criteria** | **Exclusion Criteria** |
| Population | Patients with relapsed/refractory solid tumours, any age, any prior therapies | Patients with haematological malignancies |
| Intervention | Lymphodepletion prior to T cell receptor (TCR) engineered T cell therapy (any construct, any dose) including   - chemotherapy (intravenous only) - radiotherapy - no lymphodepletion, but this must be specified | Lymphodepletion prior to   - CAR-T therapy - TIL therapy   Lymphodepletion not detailed (see Primary Outcome below)  Lymphodepletion chemotherapy route other than intravenous e.g., intra-tumoural |
| Comparator(s) | Studies with or without a comparator considered for inclusion | Not applicable |
| Outcome(s) | **Primary Outcome**  Details of, or specified absence of, lymphodepleting practices prior to TCR-engineered T cell therapy in patients with solid tumours  **Secondary Outcomes**  Studies with no safety or efficacy outcomes reported will be included in the review, and described descriptively, but excluded from safety or efficacy data analyses, and studies will be assessed for risk of bias | The presence or absence of lymphodepleting practices is not specified  If lymphodepleting practices are used, details (name of chemotherapy, radiotherapy, dose, frequency, total dose, time interval prior to TCR-T cell infusion) are not provided |
| Timing | No limits on follow up timings for safety or efficacy outcomes, due to the predicted wide variability in reported data | Not applicable |
| Study Design | Original prospective, interventional published and unpublished clinical studies   - +/-randomised - +/-controlled - single or multi-centre - > 2 patients dosed - any phase of clinical trial - articles and abstracts | - Preclinical *in vitro* and *in vivo* or human *ex vivo* studies - non-original data (narrative reviews, commentaries, letters, editorials, errata) - single case reports or ≤2 patients dosed - cost benefit analyses - language other than English |

# **Supplementary Table 3.**

# **Modified Institute of Health Economics (IHE) Risk of Bias Tool for case series studies (Quality Assessment)**

(Grigor et al., 2019, Guo et al., 2016)

| **Question Text** | **Answer Text** |
| --- | --- |
| **Study Objective**   1. Was the hypothesis/aims/objective of the study stated?   Yes- clearly stated (includes patients, intervention and outcome)  Partial/Unclear – only 1 or 2 of patients, intervention or outcome included  No – not stated | Yes  Unclear  No |
| **Study Design**   1. Was the study conducted prospectively?   Yes - clearly stated that the study was conducted prospectively  Partial/ unclear - Unclear or no information was provided  No - clearly stated it was a retrospective study | Yes  Unclear  No |
| 1. Were patients from more than one centre?   Yes - Patients were from more than one centre  Partial/ unclear - Unclear where the patients came from  No - Patients were from a single centre. | Yes  Unclear  No |
| 1. Were patients recruited consecutively?   Yes - a clear statement or clear from the context that patients were recruited consecutively  Partial/ unclear - No information was provided about the method used to recruit patients in the study.  No - clearly stated that patients were not recruited consecutively or patients were recruited based on other criteria such as access to intervention. | Yes  Unclear  No |
| **Study Population**   1. Were the eligibility criteria (i.e. inclusion and exclusion criteria) for   entry into the study clearly stated?  Yes - both inclusion and exclusion criteria were reported  Partial/ unclear - either inclusion OR exclusion were reported  No - either inclusion nor exclusion criteria were reported. | Yes  Unclear  No |
| 1. Were the characteristics of the patients included in the study   described?  Relevant characteristics: number of patients, age, sex, cancer type, prior therapies  Yes - All of the relevant patient characteristics were reported  Partial/ unclear – some, but not all, of the relevant characteristics were reported  No – Only patient numbers were reported | Yes  Unclear  No |
| 1. Did patients enter the study at a similar point in the disease?   Yes - The paper states that enrolled patients have relapsed/  refractory disease  Partial/ unclear - There was no baseline information on patients'  characteristics to make a judgment.  No - There was a wide range in the severity of the disease at baseline | Yes  Unclear  No |
| **Intervention and co-intervention**   1. Was the intervention of interest described?   Relevant characteristics: each component of lymphodepleting therapy described (chemotherapy and/or radiotherapy), including dose, frequency, timing prior to TCR-T cell infusion  Yes - All the relevant characteristics of the intervention were reported.  Partial/unclear - Some of the relevant characteristics of the intervention were reported.  No - None of the relevant characteristics of the intervention were reported. | Yes  Unclear  No |
| 1. Were additional interventions clearly described? i.e. TCR- T cell   therapy (T-cell origin, TCR target, dose, repeat doses), IL-2 administration, dendritic cell vaccination, antigen vaccination, other  Yes = All of the most relevant characteristics (type, dose, frequency  administration, duration) of the co-interventions were reported  Partial/ unclear - Some but not all of the most relevant characteristics of the co-intervention were reported.  No - No information about the co-intervention was provided; or only the name of the co-intervention was mentioned. | Yes  Unclear  No |
| **Outcome Measures**   1. Were relevant outcome measures established a priori in the introduction or methods section?   Yes - All relevant outcome measures were stated.  Partial/ unclear - Some, but not all of the relevant outcome measures were stated.  No - None of the relevant outcome measures were stated. | Yes  Unclear  No |
| 1. Were outcome assessors blinded to the intervention that patients received? i.e. did the study have 'independent outcome assessors'?   Yes - The outcomes were assessed by individuals who were not aware of patient intervention.  Partial/ unclear - The study did not report whether the outcome  assessors were aware of the intervention.  No - It was clearly stated or obvious from the context that  individuals assessing outcomes were unblinded. | Yes  Unclear  No |
| 1. Were the relevant outcomes measured using appropriate objective   or subjective methods?  Yes – Safety; adverse events listed by NCI CTCAE grade, efficacy - response rate by RECIST1.1 criteria  Partial/ unclear - 1 appropriate method or no methods listed for measurement of safety or efficacy  No – inappropriate methods were used to measure both safety and efficacy | Yes  Unclear  No |
| 1. Were the relevant outcome measures made before and after the   intervention?  Yes - The relevant outcome measures were made pre- and post- intervention  Partial/ unclear - The study did not report when the outcome  measures were made.  No - The outcome measures were only made post-intervention. | Yes  Unclear  No |
| 1. The study does not perform selective outcome reporting.   Yes - All of the study's outcomes of interest were prespecified and stated in the methods section and all were reported  Partial/ unclear – Some of the study's outcomes of interest were prespecified and stated in the methods section and some were reported  No- None of the study's reported outcomes of interest were prespecified and stated in the methods section and/or the outcomes of interest prespecified and stated in the methods section were not reported | Yes  Unclear  No |
| 1. Were details of the statistical tests reported?   Yes - The statistical tests used were reported in the study or if the data was reported descriptively with no statistical tests, this was specified  Partial/unclear - Statistical tests only partially described or reported  elsewhere (e.g previous paper, or protocol).  No - The statistical tests were not described in the study. | Yes  Unclear  No |
| 1. Was the follow-up period reported?   Yes - follow-up period was reported for both safety and efficacy  Partial/Unclear – follow-up period reported for either safety or efficacy  No - Follow-up period was not reported for any outcomes | Yes  Unclear  No |
| 1. Did the study provide estimates of random variability in the data analysis of relevant outcomes?   Yes - Estimates of the random variability (e.g. SE, SD, CI) were  reported for all relevant outcomes and/or could be calculated from the raw data.  Partial/ unclear - Estimates if the random variability were reported for some, but not all relevant outcomes.  No - Estimates of the random variability were not reported for any  of the relevant outcomes. | Yes  Unclear  No |
| 1. Were the adverse events reported?   Includes: cytopaenias, bone marrow aplasia, infection, neurotoxicity, cytokine release syndrome  Yes - All adverse events were reported or all CTC grade ≥3 were reported (if prespecified in methods)  Partial/ unclear - Unclear if all the adverse events were reported.  No - No information about adverse events reported | Yes  Unclear  No |
| 1. Were both competing interests and sources of support for the study   reported?  Yes - Both competing interests and sources of support (financial or  other) received for the study were reported; or the absence of  support was acknowledged.  Partial/ unclear - Either the competing interest or source of support  was reported.  No - Neither competing interests nor sources of support were  reported. | Yes  Unclear  No |

### Supplementary Table 4. TCR-T cell variables, Lymphodepletion Regimens (Primary Intervention) and additional therapies

| **Articles**  **First Author Year** | **TCR Target** | **Transfection**  **method** | **TCR-T cell dose**  **Median (range)** | **Additional Therapy**  **(IL-2 dose in IU/kg)** | **Lymphodepletion**  **Dose per day x no of days**  **Cyc (mg/kg, stated if mg/m^2^)**  **Flu (mg/m^2^)** | **Lymphodepletion**  **Total Dose(s)**  **Cyc (mg/kg, stated if mg/m^2^)**  **Flu (mg/m^2^)** | **Timing of LD**  **(TCR-T cell infusion day 0)** |
| --- | --- | --- | --- | --- | --- | --- | --- |
| Nagarsheth 2021 | HPV-16 E7 | γ-retrovirus | Chrt 1 1x10^9^  Chrt 2 10x10^9^  Chrt 3 100x10^9^ | IL-2 720,000 tds | 1.Cyc 30^x2 or 2. Cyc 60^x2  Flu 25 x 5 | 1.Cyc 60^ or 2. Cyc 120^  Flu 125 | -7, -6  -7 to -3 |
| D’Angelo 2021^##^ | NY-ESO-1 | Lentivirus | NR (1 - 8 × 10^9^ ) | None | Cyc 900 mg/m^2^ x 3  Flu 30 x 4 | Cyc 2700 mg/m^2^  (75 mg/kg)  Flu 120 | -7 to -4 |
| D’Angelo 2020*  (Updated data for D’Angelo 2018, Ramachandran 2019) | NY-ESO-1 | Lentivirus | See Ramachandran 2019 and D’Angelo 2018 | See Ramachandran 2019 and D’Angelo 2018 | See Ramachandran 2019 and D’Angelo 2018 | See Ramachandran 2019 and D’Angelo 2018 | See Ramachandran 2019 and D’Angelo 2018 |
| Hong D. 2020 | MAGE-A4 | Lentivirus | NR (1-5.7 x 10^9)^ | None | Cyc 600 mg/m^2^ x 3  Flu 30 x 4 | Cyc 1800 mg/m^2^  (50 mg/kg)  Flu 120 | NR |
| Nowicki 2019 | NY-ESO-1 | Retrovirus | 1x10^9^  (7.7x10^8^-1x10^9^) | DC vaccination  +/- ipilimumab Q3w  IL2 500,000 IU/m2 bd 14 d | Cyc 60 x 2  Flu 25 x 4 | Cyc 120  Flu 100 | -5, -4  -4 to -1 |
| Doran 2019 | HPV-16 E6 | γ-retrovirus | 105 x 10^9^  (1 - 170 x10^9^) | IL-2 720,000 tds | Cyc 60 x 2  Flu 25 x 5 | Cyc 120  Flu 125 | -7, -6  -5 to -1 |
| Ramachandran* 2019  (4 Cohorts,  See D’Angelo 2020 abstract, D’Angelo 2018) | NY-ESO-1 | Lentivirus | 2.67 × 10^9^  (NR) | G-CSF daily from d+2^#^  G-CSF daily from d+2^#^  G-CSF 24 hr post last Cyc dose^#^ | 1+2. Cyc 1800 mg/m^2^ x 2 (50mg/kg)  Flu 30 x 4 | 1+2.Cyc 3600 mg/m^2^  (100 mg/kg)  Flu 120 | -2, -1  -4 to -1 |
|  |  |  |  |  | 3. Cyc 1800 mg/m^2^ x 2 | 3. Cyc 3600 mg/m^2^  (100 mg/kg) | -3, -2 |
|  |  |  |  |  | 4. Cyc 600 mg/m^2^ x 3  Flu 30 × 3 | 4. Cyc 1800 mg/m^2^  (50 mg/kg)  Flu 90 | -7, -6, -5  -7, -6, -5 |
| Hattori 2019 | NY-ESO-1 | Retrovirus | NR (5 x10^8^ - 5 x10^9^) | None | Cyc 750 mg/m^2^ x 2 | Cyc 1500 mg/m^2^  (40 mg/kg) | NR |
| Butler 2019 | NY-ESO-1 | Retrovirus | 5 x 10^9^  (2.1-5 x 10^9^) | None | Cyc 750 mg/m^2^ x 2 | Cyc 1500 mg/m^2^  (40 mg/kg) | -3, -2 |
| D’Angelo 2018*  (Cohort 1, see D’Angelo 2020) | NY-ESO-1 | Lentivirus | 3.6 x 10^9^  (0.45–14.4 x 10^9^) | G-CSF daily from d+2^#^ | Cyc 1800 mg/m^2^ x 2  (50mg/kg)  Flu 30 x 4 | Cyc 3600 mg/m^2^  (100 mg/kg)  Flu 120 | -2, -1  -4 to -1 |
| Moore 2018 | Tyrosinase | Lentivirus | 2.06 x 10^8^  (2.0 -2.7 x 10^8^) | IL-2 72,000 (sic) tds  D +1 to +7 | Cyc 60 x 2  Flu 25 x 5 | Cyc 120  Flu 125 | -7, -6  -5 to -1 |
| Stadanlick 2018^##^ | NY-ESO-1 | Lentivirus | NR (1 - 5.7 x 10^9^) | None | Cyc 600 mg/m^2^ x 3  Flu 30 x 3 | Cyc 1800 mg/m^2^  (50 mg/kg)  Flu 90 | -7 to -5 |
| Hong D. 2018 | MAGE-A4 | Lentivirus | 0.1 x 10^9^ (all patients) | None | Cyc 600 mg/m^2^ x 3  Flu 30 x 3 | Cyc 1800 mg/m^2^  (50 mg/kg)  Flu 90 | -7, -6, -5 |
| Lu 2017 | MAGE-A3 | γ-retrovirus | 1. Cryopreserved,  0.65 x 10^9^  (0.01-30 x10^9)^  2. Fresh  100 x10^9^(60-123 x10^9^) | IL-2 720,000 tds | Cyc 60 x 2  Flu 25 x 5 | Cyc 120  Flu 125 | -7, -6  -5 to -1 |
| Kageyama 2015 | MAGE-A4 | Retrovirus | 1.13 x 10^8^  (1.24 x 10^7^ - 9.75 x10^8^) | MAGE 4 peptide vaccination d14, 28 | None | 0 | NA |
| Robbins 2015 | NY-ESO-1 | Retrovirus | 5.5 x 10^10^  (0.9–13 x 10^10^ ) | IL-2 720,000 tds  1 cohort received  AVIPOX-ESO vaccine | Cyc 60 x 2  Flu 25 x 5 | Cyc 120  Flu 125 | -7, -6  -5 to -1 |
| Chodon 2013 | MART-1 | Retrovirus | Cryopreserved  1 x 10^9^  (0.6 - 4.8 x 10^9^)  Fresh 3.93  (3.9-4.41 x 10^9)^ | IL-2 600,000 tds  DC vaccination | Cyc 60 x 2  Flu 25 x 5  (Flu 25 x 4 chrt 2) | Cyc 120  Flu 125  (100 chrt 2) | -7, -6  -5 to -1 |
| Morgan 2013 | MAGE-A3 | Lentivirus | 41 x10^9^  (28-79 x 10^9^) | IL-2 720,000 tds | Cyc 60 x 2  Flu 25 x 5 | Cyc 120  Flu 125 | NR |
| Hong J. 2010 | Gp100, MART-1 | Retrovirus | NR  (2.7 - 112 x 10^9^) | IL-2 720,000 tds | Cyc 60 x 2  Flu 25 x 5  (2 pts + TBI 6 Gy) | Cyc 120  Flu 125  (2 pts + TBI 6 Gy) | -7, -6  -5 to-1  -3 to-1 |

*D’Angelo 2020 abstract contains updated dosing, safety and efficacy data for D’Angelo 2018 and Ramachandran 2019

^Cyclophosphamide dose was chosen by investigators based on bone marrow reserve and comorbidities

^#^Data for the use of additional G-CSF therapy was retrieved from the full protocol published in ClinicalTrials.gov <https://clinicaltrials.gov/ct2/show/NCT01343043>
##D’Angelo 2021 has updated data for cohort 2, Stadanlick 2018 has more details for cohort 1

TCR - T cell receptor, IL-2 - Interleukin-2, G-CSF - Granulocyte colony stimulating factor, LD – Lymphodepletion, Chrt – Cohort, DC- Dendritic cell, Cyc – cyclophopsphamide, Flu- fludarabine , Tds - ter die sumendum, three times daily, TBI - Total body irradiation, Gy – Gray, NA – not applicable, NR – not reported

### Supplementary Table 5. Safety Outcomes - Adverse Event Rates for Cytopaenias and Bone Marrow Aplasia, Grade ≥3

| **Articles**  **First Author Year (cohort)** | **Total Dose Cyc/Flu** | **Febrile Neutropaenia** | **Neutropaenia** | **Lymphopaenia** | **Anaemia** | **Thrombocytopaenia** | **Bone Marrow Aplasia** | |
| --- | --- | --- | --- | --- | --- | --- | --- | --- |
| Nagarsheth 2021 (2) | 120/125 | 0.67 | 1.00 | 1.00 | 1.00 | 0.83 | Red cell aplasia 0.17 | |
| Doran 2019 | 120/125 | 0.38 | 1.00 | 1.00 | 0.85 | 1.00 | 0 | |
| Moore 2018 | 120/125 | 0.67 | 0.67 | 1.00 | NR | 1.00 | 0 | |
| Lu 2017 | 120/125 | A | A | A | A | A | 0 | |
| Robbins 2015^a^ | 120/125 | 0.76 | 1.00 | 1.00 | 0.82 | 0.40 | 0 | |
| Chodon 2013 | 120/125 | B | B | B | B | B | 0.15 | |
| Morgan 2013^$^ | 120/125 | 0.78 | 1.00 | 1.00 | 1.00 | 1.00 | 0 | |
| Hong J. 2010 | 120/125 | NR | NR | NR | NR | NR | NR | |
| Nowicki 2019 | 120/100 | 0.50 | 0.80 | 0.70 | 0.60 | 0.80 | 0.10 | |
| D'Angelo 2018* | 100/120 | 0.33 | 0.83 | 1.00 | 0.83 | 0.67 | 0 | |
| Ramachandran 2019 (2)* | 100/120 | 0.31 | 0.85 | 0.62 | 0.85 | 0.85 | 0 | |
| Ramachandran 2019 (3)* | 100/0 | 0 | 0.80 | 0.60 | 0.60 | 0.80 | 0 | |
| Nagarsheth 2021 (1) | 60/125 | 0.67 | 1.00 | 1.00 | 1.00 | 0.50 | 0 | |
| Ramachandran 2019 (4)* | 50/90 | 0.40 | 0.67 | 0.4 | 0.47 | 0.47 | 0 | |
| Kageyama 2015 | 0 | 0 | 0 | 0 | 0 | 0 | 0 | |
| **Abstracts** |  | | | | | | | |
| D'Angelo 2021 (2)^#^ | 75/120 | C | C | C | C | C | | 0 |
| Stadanlick 2018 (1)^#^ | 50/90 | NR | 0.63 | 0.75 | 0.13 | 0.38 | | 0 |
| Hong D. 2018 | 50/90 | NR | NR | NR | NR | NR | | 0 |
| Hong D. 2020 | 50/120 | NR | NR | NR | NR | NR | | 0 |
| Butler 2019 | 40/0 | NR | NR | NR | NR | NR | | 0 |
| Hattori 2019 | 40/0 | NR | NR | NR | NR | NR | | NR |

A - ‘All patients had the expected transient grade 3/4 AEs from nonmyeloablative chemotherapy regimen and high-dose IL-2’, B – ‘No unanticipated serious toxicities in the first 8 patients other than those expected from conditioning chemotherapy + high dose IL-2’, C - ‘Treatment-emergent cytopenias occurred in all patients’

* Updated data from D’Angelo 2020 abstract populates safety data for 4 cohorts for D’Angelo 2018 (1) and Ramachandran 2019 (3), therefore D’Angelo 2020 not included

# D’Angelo 2021 reports data for cohort 2, Stadanlick 2018 reports data for cohort 1, ^$^ Safety data retrieved from ClinicalTrials.gov <https://clinicaltrials.gov/ct2/show/results/NCT01273181>

^a^ Updated data retrieved from ClinicalTrials.gov<https://clinicaltrials.gov/ct2/show/results/NCT00670748?id=NCT00670748&draw=2&rank=1>

### Supplementary Table 6. Safety Outcomes (2) - Adverse Event Rates of Cytokine Release Syndrome, Neurotoxicity, Infection and Graft vs Host Disease

| **Articles**  **First Author Year (cohort)** | **Total Dose**  **Cyc/Flu** | **Cytokine Release Syndrome (CRS)**  **All grades (Grade ≥3)** | **Neurotoxicity**  **All grades (Grade ≥3)** | **Infection**  **All grades (Grade ≥3)** | **Graft versus**  **Host Disease** |
| --- | --- | --- | --- | --- | --- |
| Nagarsheth 2021 (2) | 120/125 | NR | 0 | 0 | NR |
| Doran 2019 | 120/125 | 0 | 0 | 0.31 (0.31) | NR |
| Moore 2018 | 120/125 | 0 | 0 | 0.33 (0.33) | NR |
| Lu 2017 | 120/125 | NR | 0.06 (0.06) | NR | NR |
| Robbins 2015 | 120/125 | 0 | 0 | 0 | NR |
| Chodon 2013 | 120/125 | NR | NR | NR | NR |
| Morgan 2013 | 120/125 | 0 | 0.33 (0.33) | 0.56 (grade NR) | NR |
| Hong J. 2010 | 120/125 | NR | NR | NR | NR |
| Nowicki 2019 | 120/100 | 0.2 (0.2) | 0.1 (0.1) | 0.3 (0.3) | NR |
| D'Angelo 2018* | 100/120 | 0.42 (0.17) | 0 | NR | NR |
| Ramachandran 2019 (2)* | 100/120 | NR per cohort | NR per cohort | NR | NR |
| Ramachandran 2019 (3)* | 100/0 | NR per cohort | NR per cohort | NR | NR |
| Nagarsheth 2021 (1) | 60/125 | NR | 0.17 (0.17) | 0.17 (0.17) | NR |
| Ramachandran 2019 (4)* | 50/90 | NR per cohort | NR per cohort | NR | NR |
| Kageyama 2015 | 0 | 0 | 0 | 0 | NR |
| **Abstracts** |  | | | | |
| D'Angelo 2021 (2)^#^ | 75/120 | 1.00 (0.3) | 0 | NR | 0 |
| Stadanlick 2018 (1)^#^ | 50/90 | 0.17 (0.17) | 0 | NR | NR |
| Hong D. 2018 | 50/90 | 0.33 (0) | 0.33 (0) | NR | NR |
| Hong D. 2020 | 50/120 | NR | NR | NR | NR |
| Butler 2019 | 40/0 | 0.56 (0) | NR | NR | NR |
| Hattori 2019 | 40/0 | 0.33 (grade NR) | NR | NR | NR |

* Updated data from D’Angelo 2020 abstract populates safety data for 4 cohorts for D’Angelo 2018 (1) and Ramachandran 2019 (3), therefore D’Angelo 2020 not included

# D’Angelo 2021 reports data for cohort 2, Stadanlick 2018 reports data for cohor

### Supplementary Table 7. Efficacy Outcomes - Objective Response Rate (95% Confidence Intervals), Duration of Response and Overall Survival

| **Articles**  **First Author Year (cohort)** | **Total Dose Cyc/Flu (mg/kg)**  Cyc from highest to lowest | **Objective Response Rate**  **(ORR)** | **Duration of Response**  **(Months)**  Median (range) | **Overall survival**  **(Months)**  Median (range) | **Cancer Type** |
| --- | --- | --- | --- | --- | --- |
| Nagarsheth 2021 (2) | 120/125 | 0.33 | 6.5 (4-9) | NR | Vulv, cerv, anal, SCCHN |
| Doran 2019 | 120/125 | 0.17 | 4.5 (3-6) | NR | Cerv, anal, vag, SCCHN |
| Moore 2018 | 120/125 | 0.33 | 5 (n=1) | NR | Melanoma |
| Lu 2017 | 120/125 | 0.24 | 11 (4-29+) | NR | Mel, SS, osteo, BC, cerv, anal, uro, oes |
| Robbins 2015 | 120/125 | 0.58 | 9 (3-58) | 18 (1-72)^ | SS, melanoma |
| Chodon 2013 | 120/125 | 0 | NA | 8 (1-46+) | Melanoma |
| Morgan 2013 | 120/125 | 0.56 | 5 (4-15+) | NR | Melanoma |
| Hong J. 2010 | 120/125 | 0.11 | 25+(n=1) | 15 (2-25+)^ | Melanoma +brain mets. |
| Nowicki 2019 | 120/100 | 0.20 | NR (2, 50+) | 18 (1.3-5) | SS, osteo, mel, lip, NS |
| D'Angelo 2018* | 100/120 | 0.50 | 8 (4-18) | 24.3 (8.5-48.8) | Synovial sarcoma |
| Ramachandran 2019 (2)* | 100/120 | 0.31 | 2 (2-3) | 9.9 (3.9-19.6) | Synovial sarcoma |
| Ramachandran 2019 (3)* | 100/0 | 0.20 | 8 (n=1) | 19.9 (8.8-NR) | Synovial sarcoma |
| Nagarsheth 2021 (1) | 60/125 | 0.67 | 6 (3-8) | NR | Vulv, cerv, anal, SCCHN |
| Ramachandran 2019 (4)* | 50/90 | 0.27 | 4 (3.5-23.5) | NR | Synovial sarcoma |
| Kageyama 2015 | 0 | 0 | NA | NR | Oesophageal |
| **Abstracts** | | | | | |
| D'Angelo 2021 (2)^#^ | 75/120 | 0.40 | NR | NR | Myxoid/round cell liposarcoma (MRCLS) |
| Stadanlick 2018 (1)^#^ | 50/90 | 0.50 | NR | NR | Myxoid/round cell liposarcoma (MRCLS) |
| Hong D. 2018 (1) | 50/90 | NR | NR | NR | MAGE-A4 positive tumours |
| Hong D. 2020 | 50/120 | 0.40 | NR | NR | Ov, SCCHN, MRCLS, oes |
| Butler 2019 | 40/0 | 0.20 | NR | NR | Endom, ov, SS, mel |
| Hattori 2019 | 40/0 | 0.33 | NR | NR | Synovial sarcoma |

^Median overall survival estimated from Kaplan-Meier plots, for Robbins 2015, the mean median OS data for SS(21 m) and mel(15m) is shown, OS range includes shortest and longest values for either SS or mel

*Updated efficacy data from D’Angelo 2020 abstract used to populate data for the 4 cohorts for D’Angelo 2018(1) and Ramachandran 2019 (3), D’Angelo 2020 not included

# D’Angelo 2021 reports data for cohort 2, Stadanlick 2018 reports data for cohort 1, NA – not applicable, NR – not reported

### Supplementary Figure 1. Risk of Bias Graph; Review author’s judgement regarding each risk of bias item, presented as percentages across all included studies


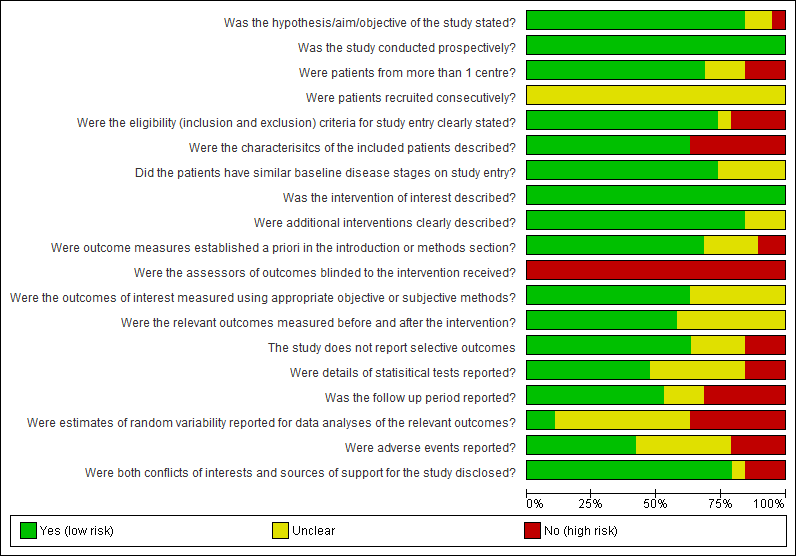


Graphs prepared by Kate Owen using Review Manager (RevMan) [Computer Program] Version 5.4. The Cochrane Collaboration, 2020.

### Supplementary Figure 2. Risk of Bias Graph; Summary of Review author’s judgement regarding each risk of bias item for Individual Studies (Internal validity)


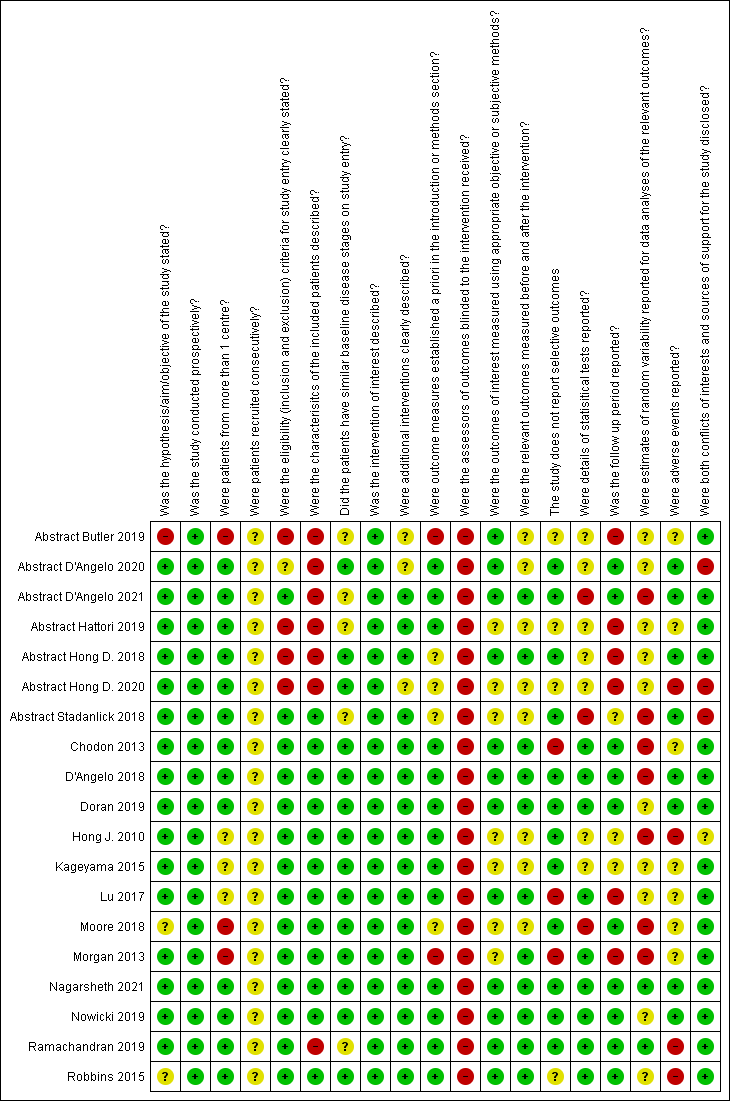


### Supplementary Figure 3: The pooled objective response rate (ORR) according to high and low dose of LD regimen.

Random effect model was applied


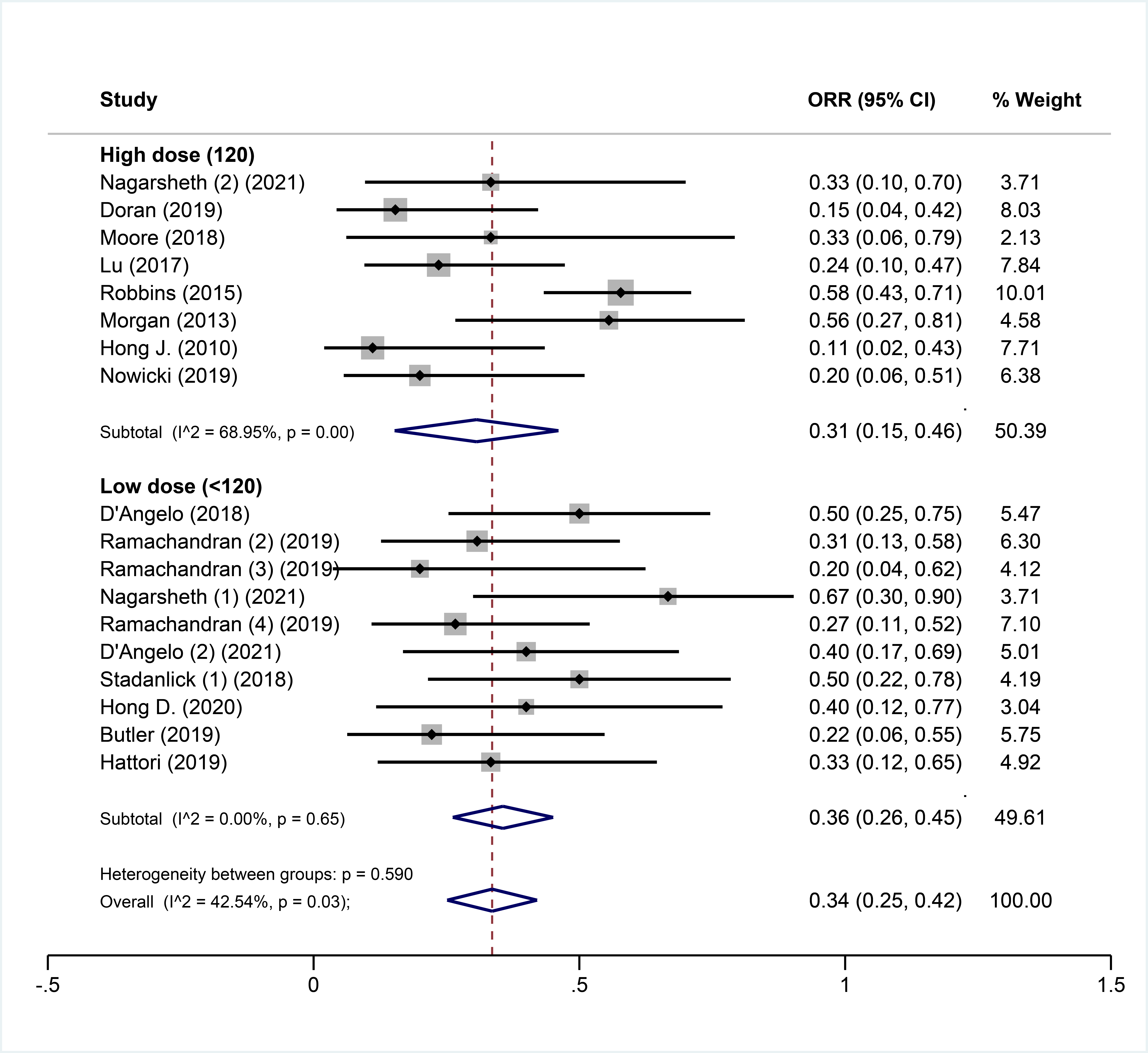

Supplement: Supplementary file 1 — Supplementary file1 (DOCX 2943 kb) [file 262_2022_3287_MOESM1_ESM.docx]
